# Double J stent combined with pyelostomy tube in pediatric laparoscopic pyeloplasty: a 5-year clinical experience in a single center

**DOI:** 10.1186/s12894-023-01351-1

**Published:** 2023-11-08

**Authors:** Zhiqiang Chen, Yunjin Wang, Changwei Wu, Hong Chen, Xu Cui, Chaoming Zhou

**Affiliations:** Fujian Children’s Hospital (Fujian Branch of Shanghai Children’s Medical Center), College of Clinical Medicine for Obstetrics & Gynecology and Pediatrics, Fujian Medical University, Fuzhou, China

**Keywords:** Ureteropelvic junction obstruction(UPJO), Laparoscopic pyeloplasty (LP), Stents, Pyelostomy tube, Complications

## Abstract

**Objective:**

To compare the outcome of using a double J (DJ) stent combined with pyelostomy tube with a DJ stent alone in laparoscopic pyeloplasty (LP) for pediatric ureteropelvic junction obstruction (UPJO).

**Methods:**

A retrospective review of all patients with UPJO treated with LP between January 2017 and November 2021 was conducted in our center. According to different postoperative drainage methods patients were divided into a DJ stent group (52 cases) and a DJ stent combined with pyelostomy tube group (combination group, 41 cases). Operative time, bleeding volume, perirenal drainage stent removal time, postoperative hospital stay, postoperative complications, and renal function recovery were compared between the two groups. Renal ultrasound and diuretic renogram (DR) were used for preoperative and postoperative follow-up.

**Results:**

A total of 52 patients were in the DJ stent group and 41 patients in the combination group. The mean hospital stay was 6.46 ± 2.66 days in the DJ stent group and 5.22 ± 1.63 days in the combination group (p < 0.05). Postoperative complications developed in 14 out of 52 patients in the DJ stent group (26.9%), while complications developed in 8 out of 41 patients in the combination group (19.5%) (p > 0.05). Non-catheter-related complications developed in 10/52 patients in the DJ stent group (19.2%) and only 1/41 patients in the combination group (2.4%) (p < 0.05). The renal function and renal cortex thickness in both groups were improved.

**Conclusion:**

Both the DJ stent drainage and the DJ stent combined with pyelostomy drainage are safe and effective. We should fully consider the patient’s preoperative and intraoperative conditions and choose appropriate drainage methods. A DJ stent combined with pyelostomy tube can reduce non-catheter related complications, facilitate postoperative recovery, and the hospital stay was significantly shorter than the DJ stent group. However, it is necessary to pay attention to the nursing treatment of the pyelostomy tube and guard against the occurrence of pyelostomy tube shedding.

## Introduction and objective

Pediatric ureteropelvic junction obstruction (UPJO) is one of the common causes of hydronephrosis in children, and the Anderson Hynes dismembered pyeloplasty is the gold standard for the treatment of UPJO. Since Peters [1] first reported laparoscopic pyeloplasty (LP) in children in 1995, laparoscopic treatment of hydronephrosis has become widely used. Compared with traditional open surgery, laparoscopic surgery has the advantages of being minimally invasive, producing a small incision, and leaving a better cosmetic appearance, and is now a well-established treatment for UPJO [2]. Although robot-assisted laparoscopic pyeloplasty.

(RALP) has started to be performed abroad, there is still controversy over whether its overall efficacy is superior to LP. Currently, RALP is still in its infancy in pediatric urology in China, so LP is still the most mainstream treatment option [3–5]. However, it is still controversial whether to use drainage and what kind of drainage should be used during pyeloplasty. Although some authors [6, 7] had described the safety and effectiveness of stentless drainage, most prefer drainage and believe that stents play an important role in supporting the anastomotic suture and draining urine and can reduce the incidence of anastomotic stenosis and urine leakage after surgery. Drainage is mainly divided into two modes; external drainage [8–10] and internal stent drainage [11–14].

Years of research have shown the effectiveness of internal and external drainage can still present a variety of complications. Questions remain whether there are other drainage methods that can effectively reduce the incidence of complications. As far as we know, there are few studies on the clinical efficacy of combined drainage mode after LP for hydronephrosis in children. The purpose of this study was to compare the clinical feasibility, advantages, and disadvantages of using a double J (DJ) stent combined with pyelostomy tube drainage compared to DJ stent drainage alone in LP.

## Materials and methods

### Selection criteria and preoperative evaluation

We retrospectively analyzed the clinical data of children who had undergone LP for treatment of UPJO in our center from January 2017 to November 2021. Our study compared the advantages and disadvantages and clinical feasibility of using a DJ stent combined with pyelostomy drainage with DJ stent drainage alone.

A total of 93 children were included in this study, and all of them underwent renal ultrasound, and diuretic renogram before surgery. Voiding cystourethrography was performed in patients with preoperative urinary infection. The degree of hydronephrosis was classified according to the fetal urology (SFU) grading system [15]. The degree of pyelocaliectasis was graded as 0–4 according to the classification scheme of the Society for Fetal Urology (0, normal kidney with intact renal sinus; 1, slightly dilated renal pelvis without caliectasis; 2, moderately dilated renal pelvis with mild caliectasis; 3, large renal pelvis and dilated calices; 4, large renal pelvis with large dilated calices).

According to the different drainage methods the patients were divided into two groups: DJ stent group (n = 42) and DJ stent combined with pyelostomy tube group (the combination group, n = 38). The clinical data of all children is shown in Table 1. There was no significant difference in age, sex, side, and degree of hydronephrosis between the two groups. Patients who underwent laparoscopic surgery for unilateral UPJO were included. The patients with solitary kidney, vesicoureteral reflux (VUR), bilateral UPJO, other renal abnormalities, recurrence of UPJO, and those who were followed up for less than one year or lost follow-up were excluded from the study. The indications for surgery were differential renal function (DRF) < 35–40%, anterior-posterior renal pelvic diameter (APD) > 30 mm, urinary tract infection, abdominal pain, or when hydronephrosis was further aggravated during follow-up.

**Table 1:** Comparison of preoperative general data between the two groups

	DJ stent group	Combination group	T value/χ^2^ value	P value
Age (month)	30.50 ± 38.23	26.17 ± 36.09	0.556	0.580
Gender (M/F)	39/13	34/7	0.853	0.356
Side (left/right)	14/38	13/28	0.255	0.614
Degree of hydronephrosis (SFU levels 2/3/4)	3/4/45	2/3/36		1.00

Preoperative urine analysis and urine culture were performed for all patients to screen for urinary tract infection.

### Surgical procedure of pyeloplasty

All procedures were performed by laparoscopic surgeons with the same qualifications and experience with pyeloplasty surgery. **The choice of drainage method was selected by the surgeon according to the preoperative or intraoperative situation.**

DJ stent group: The operations were performed through three ports with one 5 mm trocar and two 3 mm trocars. For right-sided repairs the colon was generally reflected to expose the retroperitoneum and ureteropelvic junction, on the left a transmesenteric approach was frequently utilized, especially if the ureteropelvic junction was readily visualized. The mesentery and peritoneum were opened by approximately 1 cm with an electric hook. The antetheca of the pelvis was found, and the surrounding tissue was gradually dissected. Then, a 3 − 0 absorbable suture was placed in the upper abdomen to lift the upper edge of the antetheca of the pelvis so that the renal pelvis could be completely and exactly exposed. We removed the ureteral stenosis and made a longitudinal incision of about 2 cm in the ureter. After determining the lowest point of the renal pelvis and the lowest point of the ureteral longitudinal incision, **we inserted the guide wire through the trocars, and then inserted the DJ stent anterogradely under the guide wire guidance and sutured the anastomosis.** During the operation, we used a mini gripper that can be adjusted in all directions to manipulate the renal pelvis. This is called “adjustable suspension”, which is more convenient and allows a faster procedure, and greatly improves comfort and speed of the suturing. The perinephric drain stent, and bladder catheter were indwelling in all patients.

**Fig. 1:**
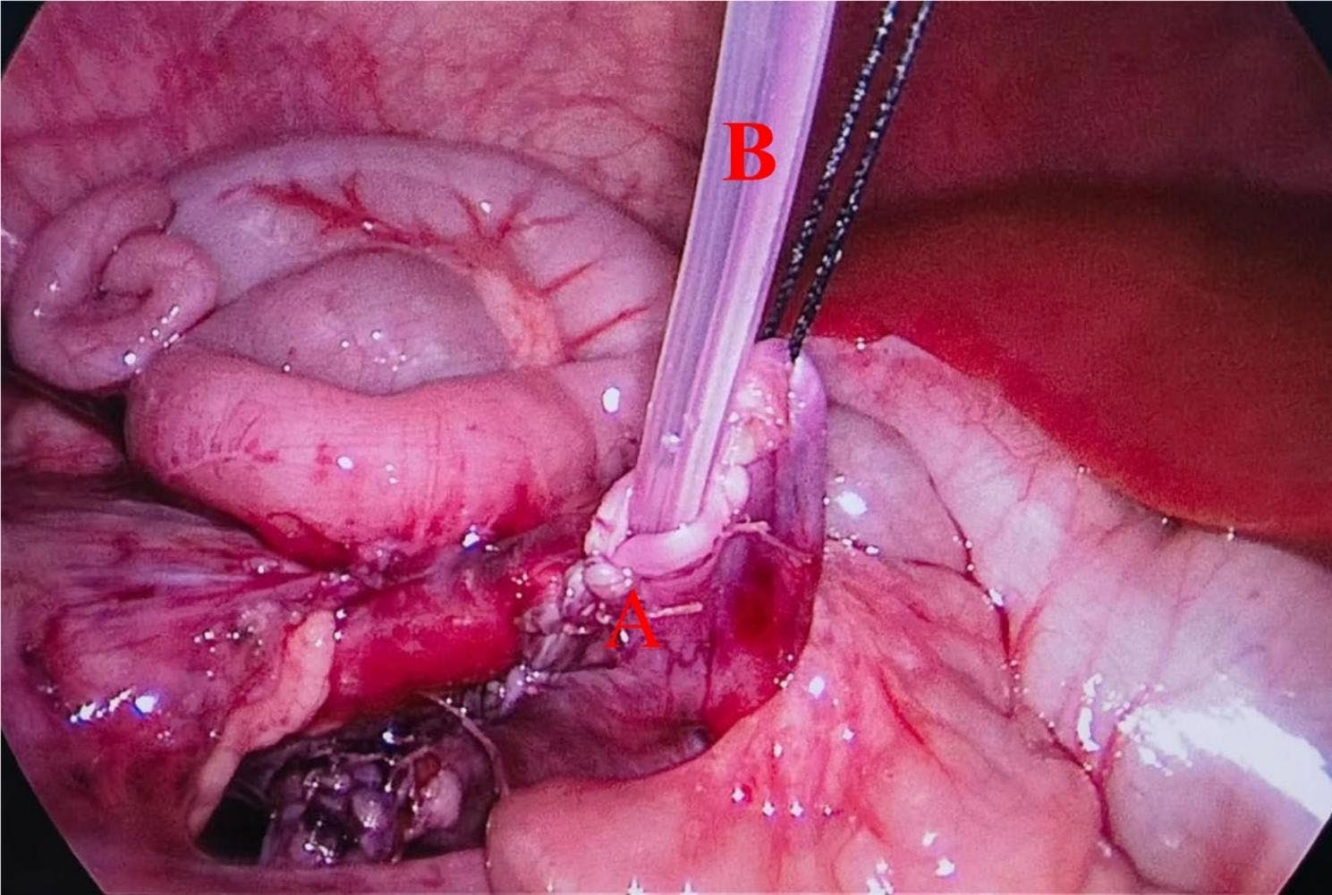
** A**: the renal pelvis **B**: the pyelostomy tube

Combination group: The previous surgical procedure was the same as DJ stent group, but the difference was the addition of a pyelostomy tube. An F8 catheter was used as the pyelostomy tube. The catheter was inserted into the abdominal cavity through an external puncture. The catheter was placed into the renal pelvis at the end of the suture to fill the water sac, and then the renal pelvis suture was completed. Figure 1 shows the position of the pyelostomy tube. The catheter was fixed to the skin with a 3 − 0 absorbable suture. The renal pelvis can be irrigated through the pyelostomy tube to reduce the formation of blood clots.

### Postoperative care

The bladder catheter was removed 1–2 days after surgery. The indication to remove the perinephric drain was the remaining output of the drainage had not increased and/or having increased by less than 10mL within 24 h. Long-term oral cefixime granules were given after discharge to prevent infection. At the 2nd, 3rd, 6th and 12th month after operation, the children were examined by ultrasonography of urinary system, and at the 6th to 12th month, the children were examined by diuretic renogram. DJ stents were scheduled for removal 2-months after surgery. In the combination group the pyelostomy tube was clamped when the DJ stent was removed. After rechecking the ultrasonography of the urinary system on the next day, the pyelostomy tube can be removed if there is no obvious increase of hydronephrosis and no discomfort such as vomiting and abdominal distension. The follow-up time was one year including both clinical and telephone follow-up to avoid missing any complications. Surgical failure was defined as a reoperation or a further increase in hydronephrosis during follow-up compared to pre-operation. The Clavien Dindo (CD) grading system was used to classify the severity of postoperative complications [16].

### Primary and secondary outcomes

The primary outcome of this retrospective study was to compare the utility and safety of two drainage methods. Additionally, the secondary outcomes was to compare the DJ stent drainage and DJ stent combined with pyelostomy tube drainage with regards to short and long term outcomes.

### Data statistics

Continuous data are presented as the mean ± standard deviation and range. Clinical parameters between the two groups were compared with the independent samples t-test. The χ^2^ test or Fisher’s test was used for categorical variables. A p value of < 0.05 was defined as statistically significant.

## Results

### Comparison of clinical parameters during and after operation

There was no significant difference between the two groups in operation time, amount of bleeding, and DJ stent extraction time. However, the indwelling time of perirenal drainage stent in the combination group(3.22 ± 0.96d)was significantly shorter than that in the DJ stent group(4.48 ± 3.07d)(p < 0.05),and the postoperative hospitalization in the combination group(5.44 ± 1.84d)was significantly shorter (p < 0.05) than that in the DJ stent group(7.15 ± 3.58d)(p < 0.05)(Table 2).

**Table 2:** Comparison of clinical parameters between the two groups during and after operation

	DJ stent group	Combination group	t value	P value
Duration of surgery (min)	157.08 ± 17.36	160.88 ± 14.71	-1.120	0.266
Amount of bleeding (ml)	7.67 ± 2.70	7.78 ± 2.17	-0.207	0.837
Postoperative hospitalization (d)	6.46 ± 2.66	5.22 ± 1.63	2.617	0.010
Indwelling time of perirenal drainage stent (d)	4.48 ± 3.07	3.22 ± 0.96	2.526	0.013
DJ stent extraction time (d)	62.77 ± 11.95	61.01 ± 11.383	0.713	0.477

### Comparison of complications

The success rate of the DJ stent group was 92.3% and that of the combination group was 100%. There were 14 cases (26.9%) of complications in the DJ stent group and 8 cases (19.5%) in the combined group, with no significant difference between the two groups(Table 3). Complications caused by the DJ stent or pyelostomy tube were defined as catheter-related complications, while others were defined as non-catheter-related complications, including urinary leakage, restenosis, bleeding, ileus, abdominal distention, and vomiting after DJ stent extraction. Among the non-catheter complications in the DJ stent group there were three cases of urinary leakage, two cases of restenosis, and one case of incisional poor healing. There was also one case of incomplete intestinal obstruction caused by urinary leakage, which recovered after stopping food and water intake and prolonging the peritoneal drainage period. Three patients had abdominal pain and vomiting after removing the DJ stent, of which two recovered after conservative treatment, and one underwent a DJ stent implantation. Two patients underwent unplanned reoperation, both of whom underwent a DJ stent implantation.

**Table 3:** Comparison of postoperative complications between the two groups

	DJ stent group (n = 52)	Combination group (n = 41)	χ^2^ value	P value	Clavien Grade
Value (%)	14 (26.9)	8 (19.5)	0.697	0.404	
Non-catheter related complications	10	1	4.693	0.03	
Urinary leakage	3	0		0.252	2I, 1II
Unplanned reoperation	2	0		0.502	2IIIb
Restenosis	2	0		0.502	2IIIb
Incisional poor healing	1	0		1.000	1I
Bleeding	0	0			
Ileus	1	0		1.000	
Abdominal distention and vomiting after DJ stent extraction	3	1		0.628	2I, 1IIIb/I
Catheter-related complications:	4	7	1.139	0.286	
Pyelonephritis	2	2		1.000	2II/2II
Stent migration	1	1		1.000	I/I
Pyelostomy tube exfoliation	0	2		0.192	2I
Intermittent flank pain or soreness	1	2		0.581	I/2I
Hematuresis	0	0			
Calculi	1	0		1.000	I

In the combination group there was only one case of abdominal pain and vomiting after removing the DJ stent. The difference between the two groups in the non-catheter related complications was statistically significant (p < 0.05).

Among the catheter-related complications, in the DJ stent group there were two cases of pyelonephritis, one of which was complicated with DJ stent calculus, one cases of DJ stent migration, and one case of abdominal pain. In the combination group there were two cases of pyelonephritis, one case of DJ stent migration, two cases of pyelostomy tube exfoliation, and two cases of abdominal pain. There was no significant difference in the total complications between the two groups.

In terms of Clavien classification, there were seven Clavien grade 1 complications, three Clavien grade 2 complications, and four Clavien grade 3 complications in the DJ stent group. The combination group had two Clavien grade 1 complications and six Clavien Grade 2 complications.

### Postoperative follow-up

Preoperative and postoperative renal function of the DJ stent group were 30.58 ± 13.59mL/min and 40.08 ± 7.07mL/min (p < 0.05), and for the combined group 29.12 ± 10.13mL/min and 39.66 ± 7.22mL/min (p < 0.05). Renal function in both groups improved after the operation. The preoperative and postoperative renal cortical thickness of the DJ stent group was 28.67 ± 14.18 mm and 52.92 ± 15.26 mm(p < 0.05), and that of the combined group was 26.95 ± 13.19 mm and 48.71 ± 14.78 mm (p < 0.05).Renal cortical thickness in both groups improved after the operation(Table 4).

**Table 4:** Postoperative recovery of the two groups

	DJ stent group (n = 53)	Combination group (n = 41)	T value	P value
Differential renal function				
Preoperative (ml/min)	30.58 ± 13.59	29.12 ± 10.13	0.571	0.569
Postoperative (ml/min)	40.08 ± 7.07	39.66 ± 7.22	0.281	0.780
P value	<0.05	<0.05		
Renal cortical thickness				
Preoperative (mm)	28.67 ± 14.18	26.95 ± 13.19	0.599	0.551
Postoperative (mm)	52.92 ± 15.26	48.71 ± 14.78	1.341	0.183
P value	<0.05	<0.05		

## Discussion

Dismembered Anderson-Hynes pyeloplasty performed via open or minimally invasive approach is the gold standard technique for the surgical treatment of UPJO in children [17, 18]. There is still discussion as to whether intraoperative drainage is needed and what drainage method to use for an anastomosis. Some practitioners advocated for a stent-less repair and do not advise any form of drainage, and no stent was placed [7]. Others argued that the stent played an important role in supporting the anastomosis and reduced the formation of urinary leakage [19]. The preference for drainage is divided into external drainage and internal drainage. No matter what kind of drainage method, the ultimate goal of drainage is to reduce the occurrence of complications and ensure the success of the operation.

This study compared the efficacy of DJ stent drainage and DJ stent drainage combined with a pyelostomy tube, so the placement of the DJ stent was an important process.

Reasonable selection of the length of the double-J tube is the basis for successful implantation. The ureter length of patients of different ages is different. Too long of a DJ stent easily causes more bladder irritation to patients, while too short of a DJ stent can easily move up or down due to the patients’ physical activity. Palmer et al. [20] provided a simple formula: ureter length = age (years) + 10 cm. Our experience is that an F3 DJ stent with a length of 120-140 mm can be used within one year of age. An F4 DJ stent with a length of 140-200 mm can be used depending on the intraoperative situation for older than 1-year old patients.

The possible risks from a DJ stent implantation include displacement, fracture, stone formation, blockage, hematuria, urinary tract infection, and low back pain. Once the ureteral stent is displaced, serious problems may occur [14]. The displacement of a DJ stent to the posterior urethra will produce serious lower urinary tract symptoms, including discomforts such as frequent urination and urgency of urination. Once the DJ stent is displaced the generated urine will cause pressure on the newly formed anastomosis, and the postoperative tissue edema may also be obstructed, resulting in further increase of postoperative hydronephrosis.

In this study, there was one case of the DJ stent ascending in the DJ stent group. Because the patient had no obvious symptoms, we found that the DJ stent was not in the bladder by renal ultrasound 2-months later. During the operation, the DJ stent was found in the lower ureter of the child through ureteroscopy, and it was diagnosed and removed successfully. This may have been caused by insufficient intraoperative experience and improper selection of the DJ stent. In the combination group there was one case of DJ stent downward movement that caused frequent and urgent urination and was automatically discharged through the urethra. However, there was no urine leakage and no special treatment.

Some studies had reported that the use of DJ stents may lead to postoperative iatrogenic vesicoureteral reflux [21], which may be related to the occurrence of postoperative pyelonephritis. In the studies of Zhu et al. and Zhang et al. [19, 22], the common postoperative complication was pyelonephritis. In our study the incidence of pyelonephritis was similar (two in each group) despite DJ stent being in place for a longer time. This may be due to the routine antibiotic prophylaxis given to all patients after discharge.

In a meta-analysis by Liu et al. in 2019 [23], the average operative time of internal drainage versus external drainage was 147 min versus 155 min for the DJ versus external PU stents. The duration of surgery for the DJ stent and the combination groups were 157.08 ± 17.36 min and 160.88 ± 14.71 min in our study. The difference was not statistically significant(p > 0.05). The hospital stay for the DJ stent group was 6.46 ± 2.66 days, and that of the combination group was 5.22 ± 1.63 days, which was similar to what has been reported in previous literature [24], but there was a statistically significant difference between the two groups (p < 0.05). The use of a pyelostomy tube combined with DJ stent reduced perirenal exudation,which allowed us to remove the perirenal drainage tube faster, which is a factor in faster discharge.

In some studies, the external drainage was from the renal parenchyma [25], which may cause damage to the renal parenchyma and aggravate bleeding. Our pyelostomy tube passed through the renal pelvis and should reduce the damage to the renal parenchyma. There was no incidence of continuous hematuria in the pyelostomy tube or catheter in this study.

Both the DJ stent drainage and the DJ stent combined with pyelostomy tube had a certain improvement effect on postoperative renal function and anterior posterior diameter of the renal pelvis. The two drainage methods were safe and effective, and the success rate of the surgery in the DJ stent group was 92.3% and that of the combination group was 100%, which was not different from previous literature reports [26]. There were 14 cases (26.9%) of complications in the DJ stent group, while only 8 cases (19.5%) in the combined stent group, all of which were Clavien grade I and II that were easy to manage. Non-stent related complications were 10 cases (19.2%) in the DJ stent group and one case (2.4%) in the combination group, and the difference was statistically significant. We believe the reason is the use of a DJ stent combined with a pyelostomy tube can significantly reduce the occurrence of urinary leakage and unplanned reoperation, as it acts as a double insurance drainage method. Park et al. [27] proposed that there was a negative correlation between the improvement of hydronephrosis and immediate postoperative obstruction, and the existence of such an obstruction, even if only temporary, would affect the postoperative outcome. This indicated the importance of adequate drainage for postoperative recovery. Due to the existence of the pyelostomy tube, even if the DJ stent is displaced, the urine can be fully drained to reduce the tension on the anastomotic stoma, which is conducive to the recovery of the anastomotic stoma after the operation. We can wash the renal pelvis to reduce the occurrence of blood clots, and we can also verify whether there is obstruction by antegrade pyelography through the pyelostomy tube [28].

In the DJ stent group, three patients developed abdominal distention and vomit after the removal of the stent, two patients recovered after conservative treatment, and one patient experienced further aggravation of hydronephrosis. Finally, their symptoms improved after the DJ stent was placed again. In the combination group one patient suffered from abdominal distension, vomit, and aggravation of hydronephrosis after removal of the DJ stent and then clamping the pyelostomy tube. The symptoms disappeared after we opened the pyelostomy tube. Finally, by prolonging the indwelling time of the pyelostomy tube to 3-months, the hydronephrosis was significantly reduced and reoperation was avoided. These cases indicate the presence of postoperative anastomotic obstruction, which mean longer hospital stays or the possibility of reoperation. This is unacceptable for children and parents, and may cause conflicts between doctors and patients, which brings great challenges to clinical work. Due to the presence of a pyelostomy tube, the likelihood of adverse reactions in children after extubation is low, and the risk of reoperation is also reduced. Meanwhile, we speculate that some patients may need more time to recover their anastomotic stomas after surgery, but this requires more research to verify.

We do not advocate blindly adding drainage tubes. We should fully consider the patient’s preoperative and intraoperative conditions and choose appropriate drainage methods. We think that for UPJO children with recurrent urinary tract infections, increased intraoperative bleeding, or complex surgical methods, the DJ stent combined with a pyelostomy tube drainage may have a good effect. However, Due to the addition of an additional drainage tube, daily care taken to clean the skin around the fistula to reduce the risk of retrograde infection. We used an F8 catheter made of silica gel. We had two cases of pyelostomy tube falling off after the operation. This is mainly because the volume of water in the catheter balloon will slowly decrease and lose its fixation function over time. Therefore, we suggest replenishing the balloon once every 2–3 weeks and regularly cleaning and disinfecting the skin around the pyelostomy tube. In addition, a pyelostomy increases the incision in the lateral abdominal wall, increases trauma, and can negatively affect the favorable cosmetic outcome of the minimally invasive procedure.

There are still several limitations to this study. First, this study was a single-center study and more studies from multiple centers are needed to further evaluate the efficacy and complications of this drainage method. Second, this study was a retrospective analysis and lacked a prospective randomized controlled arm. Third, the number of patients in this study was relatively limited, and large-scale studies are needed in the future. Fourth, the follow-up period of this study was short, and a longer follow-up is required.

## Conclusion

Both the DJ stent drainage and the DJ stent combined with pyelostomy drainage are safe and effective. We should fully consider the patient’s preoperative and intraoperative conditions and choose appropriate drainage methods. A DJ stent combined with pyelostomy tube can reduce non-catheter related complications, facilitate postoperative recovery, and the hospital stay was significantly shorter than the DJ stent group. However, it is necessary to pay attention to the nursing treatment of the pyelostomy tube and guard against the occurrence of pyelostomy tube shedding.

## Data Availability

The datasets used and/or analysed during the current study are available. from the corresponding author upon reasonable request.

## Abbreviations

UPJO: Ureteropelvic junction obstruction
LP: Laparoscopic pyeloplasty
RALP: Robot-assisted laparoscopic pyeloplasty
DJ: Double J
DR: Diuretic renogram
VUR: Vesicoureteral reflux
DRF: Differential renal function
